# Impact of Treatment Modalities on Prognosis in Patients With Renal Collecting Duct Carcinoma: A Population-Based Study

**DOI:** 10.3389/fonc.2022.810096

**Published:** 2022-04-22

**Authors:** Xiaoyuan Qian, Jinzhou Xu, Chenqian Liu, Mingliang Zhong, Senyuan Hong, Can Qian, Jianning Zhu, Jiaqiao Zhang, Shaogang Wang

**Affiliations:** ^1^ Department of Urology, Tongji Hospital, Tongji Medical College, Huazhong University of Science and Technology, Wuhan, China; ^2^ Department of Traditional Chinese Medicine and Rheumatology, Southwest Hospital, Army Military Medical University, Chongqing, China; ^3^ The Central Hospital of Wuhan, Tongji Medical College, Huazhong University of Science and Technology, Wuhan, China

**Keywords:** collecting duct carcinoma, clinical characteristics, treatment methods, prognosis, directed acyclic graphs

## Abstract

**Objective:**

Renal collecting duct carcinoma (CDC) is an extremely rare disease with few studies, and the current understanding of its prognosis is limited. We used the Surveillance, Epidemiology, and End Results (SEER) registry data to explore the prognostic factors and effect of treatment modalities on the overall survival (OS) and cancer-specific survival (CSS) in patients with CDC.

**Methods:**

Patients’ information of CDCs diagnosed by pathological examination between 2000 and 2018 was extracted from the SEER database. The Kaplan–Meier method was used to calculate OS and CSS and log-rank tests to evaluate the differences in OS and CSS. The associations between clinicopathological variables and survival outcomes were assessed with the Cox proportional hazard model. A directed acyclic graph (DAG) was drawn to recognize confounding factors and to obtain the multivariable regression model, and the impact of surgery, radiotherapy, and chemotherapy on OS and CSS was analyzed, respectively.

**Results:**

A total of 242 patients with CDC were enrolled. The median OS and CSS time were 17 and 21 months, respectively. The OS rates at 1, 2, and 5 years were 56.9%, 41.9%, and 30.0%, respectively, while the CSS rates at 1, 2, and 5 years were 60.1%, 47.5%, and 34.8%, respectively. Patients who had a large tumor size, poor pathological grade, and advanced TNM classification exhibited worse survival outcomes. Univariable and multivariable Cox regression analyses revealed that surgery, chemotherapy, T stage, N stage, and M stage were independent prognostic factors for OS and CSS. The DAG-guided multivariate Cox regression model revealed that surgery and chemotherapy improved OS and CSS.

**Conclusions:**

CDC is an exceedingly rare disease and has malignant behavior. Most patients have a high pathological grade and advanced TNM stage at diagnosis and exhibited poor survival. Resection of all visible tumors including metastatic lesions or chemotherapy can be beneficial to prognosis, while healthier benefits are less likely to receive radiotherapy. More relevant studies with larger samples are needed to verify the value of surgery and adjuvant therapy in the treatment of CDCs.

## Introduction

Collecting duct carcinoma (CDC) is a rare subtype of renal cell carcinoma (RCC), which is a malignant renal epithelial tumor, originating from the principal cells of the distal segment of the renal medullary collecting duct (1, 2). CDCs account for less than 2% of RCC and display aggressive features and poor prognosis (3–6). At the early stages of CDCs, the typical clinical symptoms and specific biomarkers are lacking and the majority of patients are found and diagnosed at the advanced TNM stage (6–8), which may result in a worse survival. Owing to the aggressive behavior of CDCs—a high incidence of distant metastasis and high pathological grade—early and accurate preoperative or postoperative diagnosis of CDCs is of importance for treatment strategies to improve the outcome of the patients with CDCs.

Robust studies about surgical treatment and prognostic factors of CDCs are lacking. Although some studies based on public databases have verified relevant prognostic factors affecting survival (4, 9, 10), the effect of different treatment modalities on outcomes of CDC was not clarified. Recently, Tang et al. reported the clinical characteristics and survival of patients with CDC. However, they failed to report oncologic outcomes for overall survival (OS) and explore the effect of different treatment methods on outcomes in detail. Therefore, we used data derived from the Surveillance, Epidemiology, and End Results (SEER) database to investigate the impact of surgery, radiotherapy, or chemotherapy, on OS and CSS respectively, using the DAG-guided multivariate Cox regression model. The clinical feature, survival outcomes, and independent prognostic factors of OS and CSS were also revealed.

## Materials and Methods

### Data Extraction

Much information on cancer incidence and survival outcomes was collected in the SEER database, and about 35% of the US population was covered (11). By running the SEER*Stat 8.3.9.2 software (https://seer.cancer.gov/seerstat/), the “Incidence-SEER Research Plus Data, 18 Registries, Nov 2020 Sub (2000-2018)” database was linked and the clinical and follow-up data of patients diagnosed with CDCs by postoperative pathology from 2000 to 2018 were retrieved and exported. According to the code of ICD-0-3: 8319/3: Collecting duct carcinoma, the patients were recruited into this study. The inclusion/exclusion criteria for patients were as follows: (1) survival data of patients were complete and available; (2) diagnosis was confirmed by histology; (3) the year of diagnosis was between 2000 and 2018; (4) laterality only contained left or right; (5) patient information came from the medical institution; (6) in terms of surgery, patients only receiving tumor destruction were not considered; and (7) cases with incomplete clinical information related to the study were excluded. Finally, a total of 242 patients with CDCs who met the criteria were recruited in this study.

### Patient Information

Information of demographic characteristics including age, sex, and race was obtained. The data of tumor features containing tumor size, TNM stage, pathological grade, and laterality of tumor were also chosen. Next, the treatment strategies of CDCs which consisted of surgery, chemotherapy, or radiotherapy were extracted. Radiotherapy and chemotherapy were categorized into “no/unknown” or “yes.” Notably, the intent of radiotherapy for these cases is unclear and a significant proportion of patients were probably given radiotherapy for palliation. Additionally, OS was calculated from the date of diagnosis confirmed to any cause of death, regarded as the primary survival outcome. Additionally, CSS was defined as the time from the date of diagnosis to the date of death caused by CDCs.

### Statistical Analysis

All statistical analyses were performed, using R 3.6.3 software (The R Foundation for Statistical Computing, Vienna, Austria). Continuous variables with a normal distribution were described as mean ± standard deviation (SD) while clinical characteristics conforming to the skewed distribution were exhibited by the median and interquartile range (IQR). Categorical variables were recorded in numbers and percentages during descriptive statistics. The Kaplan–Meier method was used to conduct survival curves, and log-rank tests were applied to compare differences between survival curves. The univariable and multivariable Cox proportional hazards regression model was used to analyze independent prognostic factors affecting OS and CSS. Moreover, under the guidance of DAG, three different multivariable regression models were constructed to explore the impact of surgery, chemotherapy, and radiotherapy on OS and CSS, respectively. *p* < 0.05 (two-sided) was considered to be statistically significant.

## Results

### Clinical Characteristics of CDCs

A total of 242 patients diagnosed with CDCs were enrolled in this study, and their baseline clinicopathological features are shown in **Supplementary Table S1**. Of the total patients, there were 71 (29.3%) females and 171 (70.7%) males. In terms of age, 161 (66.5%) of patients were less than 68 years old and 81 (33.5%) were more than 68 years old. The included population mainly consisted of 60 (24.8%) black, 166 (68.6%) white, and other races accounting for only 16 (6.6%).

The maximum tumor diameter ranged from 4.1 to 8.5 cm, and the median tumor size was 6.3 cm. According to the TNM stage system, 77 (31.8%) cases were in T1, 16 (6.6%) in T2, 121 (50.0%) in T3, and 21 (8.7%) in T4. Respectively, 94 (38.9%) and 86 (35.5%) patients had clinical lymph node metastasis and/or distant metastases at presentation. Based on the AJCC 6th edition staging system for renal carcinoma, there were 57 (23.6%) stage I, 11 (4.6%) stage II, 57 (23.6%) stage III, and 111 (45.9%) stage IV tumors, while pathological grade revealed that 3.72%, 10.7%, 38.0%, and 26.4% of the patients were in stages I, II, III, and IV disease, respectively. During the treatment, most patients (85.1%) with CDCs underwent surgical resection and the remaining patients (14.9%) received conservative treatment. However, only a few patients received radiotherapy or chemotherapy, accounting for 25 (10.3%) and 64 (26.4%), respectively.

### Influence of Clinical Factors and Treatment Methods on Survival

To investigate the impact of clinical factors and treatment methods on OS and CSS, the Kaplan–Meier method and log-rank test were used. The median follow-up time of all patients was 17.0 months (IQR: 5.0–68.0 months). The overall 1-, 2-, and 5-year survival rates were 56.9%, 41.9%, and 30.0%, respectively, and for CSS, its 1-, 2-, and 5-year survival rates were 60.1%, 47.5%, and 34.8%, respectively. Patients with larger tumor sizes had shorter survival (median OS: 11 vs. 25 months, *p* < 0.001, and median CSS: 11 vs. 32 months, *p* < 0.001) than those with smaller tumor sizes (**Supplementary Figures S1A** and **S2A**). The survival rates were also affected by pathological grade. The median survival times of grades I, II, III, and IV for OS were 134, 97, 13.5, and 17.5 months, respectively (the total *p* < 0.001; I vs. IV, *p* = 0.017; II vs. III, *p* = 0.016; II vs. IV, *p* < 0.001) (**Supplementary Figure S1B**), for CSS not reached, not reached, 15 months, and 18 months (the total *p* < 0.001; I vs. III, *p* = 0.013; I vs. IV, *p* = 0.002; II vs. III, *p* = 0.002; II vs. IV, *p* < 0.001) (**Supplementary Figure S2B**). Similarly, tumors with a high AJCC stage were not good for the survival time of CDCs. The findings demonstrated that the median survival times of stages I, II, III, and IV for OS were 115, 91, 22, and 7 months, respectively (the total p < 0.001; I vs. III, *p* < 0.001; I vs. IV, *p* < 0.001; II vs. IV, *p* < 0.001; III vs. IV, *p* < 0.001) (**Supplementary Figure S1C**), for CSS not reached, not reached, 26 months, and 7 months (the total *p* < 0.001; I vs. III, *p* < 0.001; I vs. IV, *p* < 0.001; II vs. III, *p* = 0.041; II vs. IV, *p* < 0.001; III vs. IV, *p* < 0.001) (**Supplementary Figure S2C**). Besides, patients with the late T stage had a lower survival rate than those with the early T stage. For OS, 81, 56, 11, and 10 months were respectively matched to the median survival times of T1, T2, T3, and T4 (the total *p* < 0.001; T1 vs. T3, *p* < 0.001; T1 vs. T4, *p* < 0.001; T2 vs. T3, *p* = 0.030; T2 vs. T4, *p* = 0.007) (**Supplementary Figure S1D**). For CSS, the median survival times of T1, T2, T3, and T4 were 115, 91, 15, and 10 months, respectively (the total *p* < 0.001; T1 vs. T3, *p* < 0.001; T1 vs. T4, *p* < 0.001; T2 vs. T3, *p* = 0.013; T2 vs. T4, *p* = 0.003) (**Supplementary Figure S2D**).

Lymph node metastasis can result in worse survival. The median survival times of N0, N1, and N2 for OS were 42, 7, and 9 months, respectively (the total *p* < 0.001; N0 vs. N1 *p* < 0.001; N0 vs. N2, *p* < 0.001) (**Supplementary Figure S1E**), and 95, 9, and 9 months corresponded to the median survival times of N0, N1, and N2 for CSS (the total *p* < 0.001; N0 vs. N1 *p* < 0.001; N0 vs. N2, *p* = 0.001) (**Supplementary Figure S2E**). Compared with tumors with distant metastasis, patients without distant metastasis displayed better OS (median OS:41 vs. 5 months, *p* < 0.001) (**Supplementary Figure S1F**) and CSS (median CSS: 95 vs. 6 months, *p* < 0.001) (**Supplementary Figure S2F**). Patients who underwent surgery had longer survival than those who did not (median OS: 22 vs. 4 months, *p* < 0.001, and median CSS: 27 vs. 4 months, *p* < 0.001) (**Supplementary Figures S1G** and **S2G**). Interestingly, patients who underwent radiotherapy had a worse survival than those who did not (median OS: 7 vs. 20 months, *p* < 0.001, and median CSS: 10 vs. 25 months, *p* = 0.001) (**Supplementary Figures S1H** and **S2H**). Furthermore, patients who received chemotherapy had shorter survival than those who did not (median OS: 12.5 vs. 21 months, *p* < 0.001, and median CSS: 14 vs. 32 months, *p* < 0.001) (**Supplementary Figures S1I** and **S2I**).

**Figure 1:**
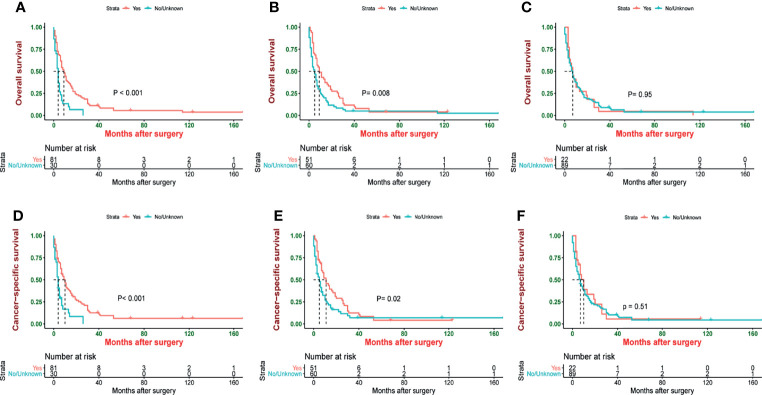
Kaplan–Meier estimate of overall survival (OS) and cancer-specific survival (CSS) stage IV patients by **(A)** surgery, **(B)** chemotherapy, **(C)** radiotherapy, **(D)** surgery, **(E)** chemotherapy, and **(F)** radiotherapy.

Since most patients receiving chemotherapy and radiotherapy were in stage IV, we included these patients for subgroup analysis. Patients who underwent surgery had better survival than those who did not (median OS: 9 vs. 4 months, *p* < 0.001, and median CSS: 10 vs. 4 months, *p* < 0.001) (**Figures 1A, D**). However, compared with a patient who did not receive chemotherapy, patients receiving chemotherapy had a longer survival (median OS: 9 vs. 5 months, *p* = 0.008, and median CSS: 11 vs. 5 months, *p* = 0.02) (**Figures 1B, E**). Meanwhile, patients who underwent radiotherapy had the same survival as those who did not undergo radiotherapy (median OS: 7 vs. 7 months, *p* = 0.95, and median CSS: 10 vs. 7 months, *p* = 0.51) (**Figures 1C, F**).

### Risk Factors for OS and CSS In Patients With CDCs

The Cox regression models were used to recognize the independent predictors of OS and CSS. By univariate analysis, the result showed that tumor size, pathological grade, AJCC stage, T stage, N stage, M stage, surgery, radiotherapy, and chemotherapy were closely related to OS and CSS (*p* < 0.05). All factors with statistical significance (*p* < 0.05) in univariate analysis were selected and submitted to multivariable Cox regression model analysis. Next, we detected multicollinearity between these variables and found that the variance inflation factors of the AJCC stage which was included in the model were more than 5. Therefore, the factor of the AJCC stage was not enrolled in the multivariable Cox regression model. In multivariate analysis, advanced T stage (T3 vs. T1 OS: HR: 1.896, 95% CI: 1.128–3.178; *p* = 0.016; CSS: HR: 2.105, 95% CI: 1.181–3.752; *p* = 0.012), N stage (N1 vs. N0: OS: HR: 2.105, 95% CI: 1.279–3.464; *p* = 0.003; CSS: HR: 2.093, 95% CI: 1.233–3.551; *p* = 0.006), M stage (OS: HR: 4.726, 95% CI: 2.843–7.856; *p* < 0.001; CSS: HR: 4.864, 95% CI: 2.850–8.299; *p* < 0.001), surgery (OS: HR: 4.526, 95% CI: 2.070–9.897; *p* < 0.001; CSS: HR: 4.844, 95% CI: 2.113–11.104; *p* < 0.001), chemotherapy (OS: HR: 3.154, 95% CI: 1.896–5.248; *p* < 0.001; CSS: HR: 2.871, 95% CI: 1.682–4.899; *p* < 0.001) remained significant prognostic factors for OS and CSS. The above findings of the Cox regression analysis of prognostic factors for OS and CSS are presented in **Tables 1**, **2**, respectively.

**Table 1:** Univariate and multivariate Cox regression analysis of the associations between clinicopathological features and OS in patients with CDCs.

Characteristics	Univariable analysis		Multivariable analysis	
	HR (95% CI)	*p*	HR (95% CI)	*p*
Age (years)	1.010 (0.999–1.022)	0.074		
Sex				
Female	Reference		Reference	
Male	1.107 (0.806–1.520)	0.529		
Race				
Black	Reference		Reference	
White	0.992 (0.712–1.383)	0.964		
Other*^a^*	0.856 (0.443–1.652)	0.642		
Tumor stage				
I	Reference			
II	0.985 (0.380–2.555)	0.975		
III	2.392 (1.475–3.878)	<0.001		
IV	6.756 (4.356–10.479)	<0.001		
Laterality				
Left	Reference		Reference	
Right	1.181 (0.885–1.576)	0.257		
Tumor size (cm)	1.001 (1.000–1.002)	0.012	1.002 (1.000–1.004)	0.074
Pathologic grade				
Grade I	Reference		Reference	
Grade II	0.945 (0.336–2.652)	0.914	0.618 (0.205–1.858)	0.391
Grade III	2.225 (0.896–5.527)	0.085	0.919 (0.340–2.481)	0.867
Grade IV	2.689 (1.071–6.755)	0.035	1.280 (0.468–3.501)	0.631
T stage				
T1	Reference		Reference	
T2	1.117 (0.564–2.214)	0.751	1.618 (0.708–3.695)	0.254
T3	2.303 (1.620–3.274)	<0.001	1.896 (1.128–3.187)	0.016
T4	3.347 (1.964–5.704)	<0.001	1.259 (0.613–2.585)	0.530
N stage				
N0	Reference		Reference	
N1	3.308 (2.284–4.791)	<0.001	2.105 (1.279–3.464)	0.003
N2	2.957 (2.026–4.318)	<0.001	1.287 (0.771–2.147)	0.335
M stage				
M0	Reference		Reference	
M1	4.684 (3.420–6.416)	<0.001	4.726 (2.843–7.856)	<0.001
Surgery				
Yes	Reference		Reference	
No	3.493 (2.391–5.104)	<0.001	4.526 (2.070–9.897)	<0.001
Radiotherapy				
Yes	Reference		Reference	
No/unknown	0.451 (0.293–0.692)	<0.001	1.125 (0.617–2.052)	0.701
Chemotherapy				
Yes	Reference		Reference	
No/unknown	0.595 (0.433–0.816)	0.001	3.154 (1.896–5.248)	<0.001

CDC, renal collecting duct carcinoma; CI, confidence interval; HR, hazard ratio; OS, overall survival.

a Other included American Indian/Alaskan Native and Asian/Pacific Islander.

**Table 2:** Univariate and multivariate Cox regression analysis of the associations between clinicopathological features and CSS in patients with CDCs.

Characteristics	Univariable analysis		Multivariable analysis	
	HR (95% CI)	*p*	HR (95% CI)	*p*
Age (years)	1.001 (0.989–1.012)	0.933		
Sex				
Female	Reference			
Male	1.320 (0.921–1.892)	0.130		
Race				
Black	Reference			
White	1.063 (0.540–2.091)	0.860		
Other*^a^*	1.103 (0.756–1.608)	0.611		
Tumor stage				
I	Reference			
II	1.027 (0.297–3.551)	0.966		
III	3.341 (1.807–6.177)	<0.001		
IV	10.768 (6.138–18.890)	<0.001		
Laterality				
Left	Reference			
Right	1.129 (0.822–1.551)	0.450		
Tumor size (cm)	1.001 (1.000–1.002)	0.003	1.002 (1.000–1.004)	0.055
Pathologic grade				
Grade I	Reference		Reference	
Grade II	2.852 (0.356–22.810)	0.323	1.814 (0.216–15.209)	0.583
Grade III	9.675 (1.340–69.870)	0.024	3.367 (0.442–25.675)	0.241
Grade IV	11.597 (1.600–84.130)	0.015	4.593 (0.600–35.182)	0.142
T stage				
T1	Reference		Reference	
T2	1.019 (0.452–2.301)	0.963	1.632 (0.632–4.209)	0.311
T3	2.658 (1.782–3.966)	<0.001	2.105 (1.181–3.752)	0.012
T4	3.804 (2.130–6.795)	<0.001	1.346 (0.621–2.918)	0.452
N stage				
N0	Reference		Reference	
N1	3.666 (2.455–5.475)	<0.001	2.093 (1.233–3.551)	0.006
N2	3.689 (2.470–5.509)	<0.001	1.407 (0.824–2.405)	0.211
M stage				
M0	Reference		Reference	
M1	5.477 (3.903–7.686)	<0.001	4.864 (2.850–8.299)	<0.001
Surgery				
Yes	Reference		Reference	
No	3.546 (2.366–5.314)	<0.001	4.844 (2.113–11.104)	<0.001
Radiotherapy				
Yes	Reference		Reference	
No/unknown	0.472 (0.296–0.753)	0.002	1.277 (0.671–2.431)	0.456
Chemotherapy				
Yes	Reference		Reference	
No/unknown	0.504 (0.361–0.702)	<0.001	2.871 (1.682–4.899)	<0.001

CDC, renal collecting duct carcinoma; CI, confidence interval; HR, hazard ratio; CSS, cancer-specific survival.

a Other included American Indian/Alaskan Native and Asian/Pacific Islander.

### Effect of Surgery, Chemotherapy, and Radiotherapy on OS and CSS

In an attempt to explore the influence of surgery, radiotherapy, and chemotherapy on OS and CSS, DAG was drawn to elucidate that the structure of the causal relation between surgery and survival outcomes (OS and CSS), chemotherapy and survival outcomes, or radiotherapy and survival outcomes, respectively, and the confounding factor in the Cox regression model were identified correctly. In the light of the relationship between each exposure factor and outcomes, a total of three DAGs were produced to direct the multivariable regression analysis model. When surgery was deemed as the main exposure factor, chemotherapy and radiotherapy as intermediate variables should be excluded from the Cox regression model (**Supplementary Figure S3**). The result of the multivariable analysis directed by DAGs showed that surgery was associated with OS (HR: 3.300, 95% CI: 1.568–6.946; *p* = 0.004) and CSS (HR: 3.398, 95% CI: 1.555–7.425; *p* = 0.002) (**Table 3**). However, chemotherapy as a confounding factor was chosen as an exposure factor, surgery was not excluded, and radiotherapy as an ancestor of outcome was excluded (**Supplementary Figure S4**). The DAGs which guided the multivariate regression model demonstrated that chemotherapy was associated with OS (HR: 2.918, 95% CI: 1.750–4.868; *p* < 0.001) and CSS (HR: 2.747: 95% CI: 1.604–4.703; *p* < 0.001) (**Table 4**). Additionally, if the exposure factor was radiotherapy, surgery seen as a confounding variable factor was also not excluded and chemotherapy as an ancestor of outcome was not included (**Supplementary Figure S5**). Finally, the findings presented that radiotherapy was not associated with OS (HR: 1.139, 95% CI: 0.613–12.115; *p* = 0.680) and CSS (HR: 1.292, 95% CI: 0.667–2.501; *p* = 0.448) (**Table 5**).

**Table 3:** DAG-guided multivariable Cox regression model analysis of causal effect of surgery on OS and CSS.

Variables	OS		CSS	
	HR (95% CI)	*p*	HR (95% CI)	*p*
Surgery				
Yes	Reference		Reference	
No	3.300 (1.568–6.946)	0.004	3.398 (1.555–7.425)	0.002
Age (years)	1.016 (1.002–1.029)	0.020	1.009 (0.996–1.023)	0.187
Tumor size (mm)	1.003 (1.001–1.005)	0.010	1.003 (1.001–1.005)	0.011
Pathologic grade				
Grade I	Reference		Reference	
Grade II	0.591 (0.197–1.773)	0.348	1.834 (0.220–15.331)	0.575
Grade III	0.958 (0.356–2.580)	0.933	3.628 (0.477–27.585)	0.213
Grade IV	1.128 (0.413–3.082)	0.814	4.120 (0.538–31.539)	0.172
T stage				
T1	Reference		Reference	
T2	1.403 (0.620–3.176)	0.416	1.400 (0.549–3.573)	0.481
T3	1.510 (0.899–2.534)	0.110	1.692 (0.954–3.001)	0.072
T4	1.1528 (0.550–2.416)	0.706	1.239 (0.559–2.746)	0.599
N stage				
N0	Reference		Reference	
N1	1.486 (0.916–2.412)	0.109	1.544 (0.928–2.570)	0.095
N2	1.156 (0.691–1.934)	0.580	1.265 (0.742–2.159)	0.388
M stage				
M0	Reference		Reference	
M1	2.779 (1.814–4.257)	<0.001	2.867 (1.836–4.477)	<0.001

CDC, renal collecting duct carcinoma; DAG, directed acyclic graphs; CI, confidence interval; HR, hazard ratio; OS, overall survival; CSS, cancer-specific survival.

**Table 4:** DAG-guided multivariable Cox regression model analysis of causal effect of chemotherapy on OS and CSS.

Variables	OS		CSS	
	HR (95% CI)	*p*	HR (95% CI)	*p*
Chemotherapy				
Yes	Reference		Reference	
No/unknown	2.918 (1.75–4.868)	<0.001	2.747 (1.604–4.703)	<0.001
Surgery				
Yes	Reference		Reference	
No	4.131 (1.971–8.659)	<0.001	4.258 (1.95–9.298)	<0.001
Age (years)	1.012 (0.998–1.025)	0.092	1.005 (0.991–1.019)	0.464
Tumor size (mm)	1.002 (1.000–1.004)	0.031	1.002 (1.000–1.004)	0.031
Pathologic grade				
Grade I	Reference		Reference	
Grade II	0.582 (0.193–1.758)	0.337	1.791 (0.213–15.045)	0.592
Grade III	0.901 (0.334–2.433)	0.838	3.400 (0.447–25.899)	0.237
Grade IV	1.285 (0.471–3.51)	0.625	4.624 (0.604–35.402)	0.140
T stage				
T1	Reference		Reference	
T2	1.596 (0.698–3.646)	0.268	1.598 (0.62–4.119)	0.332
T3	1.778 (1.057–2.991)	0.030	1.999 (1.123–3.559)	0.019
T4	1.253 (0.611–2.567)	0.538	1.329 (0.612–2.888)	0.472
N stage				
N0	Reference		Reference	
N1	2.047 (1.232–3.401)	0.006	2.069 (1.215–3.522)	0.007
N2	1.295 (0.771–2.178)	0.329	1.419 (0.827–2.436)	0.204
M stage				
M0	Reference		Reference	
M1	4.621 (2.81–7.598)	<0.001	4.694 (2.781–7.923)	<0.001

CDC, collecting duct carcinoma; DAG, directed acyclic graphs; CI, confidence interval; HR, hazard ratio; OS, overall survival; CSS, cancer-specific survival.

**Table 5:** DAG-guided multivariable Cox regression model analysis of causal effect of radiotherapy on OS and CSS.

Variables	OS		CSS	
	HR (95% CI)	*p*	HR (95% CI)	*p*
Radiotherapy				
Yes	Reference		Reference	
No/unknown	1.139 (0.6133–2.115)	0.68	1.292 (0.667–2.501)	0.448
Surgery				
Yes	Reference		Reference	
No	3.530 (1.571–7.936)	0.002	3.881 (1.654–9.105)	0.002
Age (years)	1.016 (1.003–1.029)	0.02	1.010 (0.996–1.023)	0.173
Tumor size (mm)	1.003 (1.001–1.005)	0.0115	1.003 (1.000–1.005)	0.015
Pathologic grade				
Grade I	Reference		Reference	
Grade II	0.589 (0.196–1.765)	0.344	1.816 (0.217–15.176)	0.582
Grade III	0.953 (0.354–2.566)	0.923	3.579 (0.470–27.227)	0.218
Grade IV	1.132 (0.414–3.090)	0.81	4.136 (0.540–31.667)	0.172
T stage				
T1	Reference		Reference	
T2	1.411 (0.623–3.194)	0.409	1.412 (0.554–3.603)	0.470
T3	1.529 (0.908–2.573)	0.11	1.734 (0.976–3.082)	0.061
T4	1.155 (0.552–2.417)	0.703	1.245 (0.563–2.756)	0.589
N stage				
N0	Reference		Reference	
N1	1.476 (0.907–2.402)	0.117	1.522 (0.911–2.544)	0.109
N2	1.154 (0.690–1.930)	0.585	1.261 (0.740–2.152)	0.394
M stage				
M0	Reference		Reference	
M1	2.813 (1.828–4.329)	<0.001	2.934 (1.870–4.603)	<0.001

CDC, collecting duct carcinoma; DAG, directed acyclic graphs; CI, confidence interval; HR, hazard ratio; OS, overall survival; CSS, cancer-specific survival.

## Discussion

CDC, also called Bellini duct carcinoma, is a very rare subtype of renal malignancies and presents an aggressive clinical course, with low incidence and worse outcomes (6, 10). Currently, most of the works mainly focus on a case report and case series reports and systematic studies with large sample cases are lacking. How to recognize CDC early, make the correct diagnosis, and make appropriate treatment strategies is extremely important to improve the prognosis of CDCs. Additionally, standard treatment strategies are still not available. Most patients with CDC received adjuvant radiotherapy, chemotherapy, and immunotherapy after surgery including partial nephrectomy (PN), radical nephrectomy (RN), and cytoreductive nephrectomy (CNx). There exist several questions that need to be addressed during the treatment. As we know, patients with cT1−2/N0M0 clear cell RCC receiving PN or RN can obtain equivalent oncologic outcomes (12). Doubtfully, whether patients with T1−2/N0M0 CDC receiving surgery can get similar oncologic outcomes needs to be resolved. For metastatic CDC, whether CNx benefits the patient is urgent to be illuminated. Moreover, whether all patients with CDC should receive adjuvant therapy is questioned. Here, we obtained the data from the SEER database to describe the clinical characteristics of the CDC and demonstrated its independent risk factors. Moreover, we verified the impact of different treatment methods on the survival of patients with CDC. Our findings suggested that CDCs have malignant behavior and that resection of all visible tumors or chemotherapy is significantly associated with outcomes. For patients with advanced CDC, no correlation between radiotherapy and outcomes is seen.

In the present study, we reconfirmed that CDCs showed some aggressive behavioral characteristics, and patients with CDCs had a poor prognosis. Many studies have reported that the majority of patients had high pathological grade, advanced T stage, positive lymph node, and distant metastasis at diagnosis and these factors were the independent predictor of CDCs. In our previous study (6), most patients had obvious invasive pathologic features and half of the patients had distant metastasis. They all had short survival times. Similar results can also be seen in previous studies. In a study published by Karakiewicz and his colleagues, T3 or higher accounted for 80.5%, positive node for 48.8%, and metastatic disease for 19.5% at diagnosis. In 78.0% of patients, their Fuhrman grades were grade III or higher, and the 5-year CSS for CDCs was 48% (3). Another large multi-institutional cohort from Japan revealed that more than 50% of patients had a late T stage and 97.8% of patients had a poor Fuhrman grade and that disease-specific survival was 34.3% (8). Similarly, the OS rate reduced to 26.8% in the recent work based on the SEER database (9). The authors discovered that CDC presented more often with T3 (52.8%), node-positive (40.6%), and metastatic (42.0%) diseases. An early study from 16 European and American institutions also reported that the 5-year CSS rate for CDCs was 40.3% (13). Compared to these works, our findings demonstrate the same incidence of late T stage, lymph node positive, or metastatic disease. Furthermore, the OS and CSS at 5 years were 30.0% and 34.8%, respectively, which were inferior to the survival time of RCC (14). Moreover, more than 50% of the patients with CDC died within 5 years after diagnosis and treatment and a poor prognosis was discovered. Additionally, older age, larger tumor size, late T stage, positive node, distant metastasis, poorly Fuhrman grade, and lymphovascular invasion were closely associated with worse survival outcomes (4, 10, 13), which was similar to our results. In our study, larger tumor size, advanced TNM stage, late AJCC stage, and poor pathological grade exhibited an extremely detrimental prognosis. Finally, the above factors remained independent prognosis factors for OS and CSS in the multivariable regression analysis model.

For the treatment of CDC, patients with CDC could benefit from surgical treatment and chemotherapy. When we included patients in all stages for the survival analysis, patients who received chemotherapy and radiotherapy displayed shorter survival than those who did not. However, in the next subgroup analysis, we found that patients with advanced CDC who underwent chemotherapy had longer survival than those who did not, while patients with advanced CDC who received radiotherapy had the same survival time as those who did not. The reason for this phenomenon is that for most patients receiving chemotherapy or radiotherapy, their intent of chemotherapy or radiotherapy was likely palliative and these patients themselves had a poorer prognosis than those in the earlier stages. Although no explicit treatment strategy is established, there is no doubt that surgery is still the primary treatment. Generally, patients with CDCs after surgery can obtain a longer survival (4, 10, 15). Consistent results were presented in our study. Additionally, our study revealed that advanced CDC patients benefited from surgery. In terms of surgical methods, at present, RN is suggested if tumors are suspected to be CDC before surgery for their malignant biological behavior. A minority of patients with low-stage and low-grade receiving RN did not show signs of tumor progression, suggesting that RN during the treatment of CDCs can be effective and curative (6, 16). Certainly, a few patients with early TNM stage achieved better outcomes after nephron-sparing surgery (17). In clinical practice, the surgical strategies often depend upon complicating factors such as preoperative patient status, surgical risks, survival outcomes, and distant metastasis (18). Thus, for the patient with node or distant metastasis or unable to receive CNx, adjuvant therapy may play an important role in improving the survival of CDCs. Wilson and colleagues reported that CNx combined with chemo/radiotherapy or chemo/radiotherapy alone was associated with a survival benefit over a single CNx in patients with CDCs, indicating the potential benefit for combination treatment (4). A partial response or complete remission acquired in patients receiving the therapy of chemotherapeutic agents (gemcitabine and either cisplatin or carboplatin) was discovered in previous studies (8, 18–21). Nevertheless, the only slight improvement in OS has been revealed, and progression of tumor and failure of first-line therapy was often observed in CDCs (22). Different from chemotherapy, although radiotherapy was found to play a certain role in delaying tumor progression reported in Wilson and colleagues’ study (4), the benefit from radiotherapy in the treatment of CDCs has not been unfolded in other relevant works (8, 10). In this study, most of the patients in stage IV received radiotherapy and the effect of radiotherapy on the survival of patients was not seen. Furthermore, we used DAG that can explicitly exclude irrelevant variables to enroll the real confounding factors into the Cox regression model to analyze the impact of surgery, chemotherapy, and radiotherapy, respectively, on the survival of CDCs. Finally, we found that surgery and chemotherapy were beneficial to prognosis, while healthier benefits are less likely to receive radiotherapy.

Undoubtedly, there exist several limitations in the present study. First, despite the data we used in this study from the SEER database, the number of cases included is still small. Second, this study belongs to a retrospective study and potential selection bias is inevitable. Third, chemotherapy schedule and administration time are unclear and the effect of specific chemotherapeutic drugs on the survival of CDCs remains to be studied. Fourth, the type of surgery is unknown and the role of NSS and RN in the treatment of patients with early CDCs is also folded. Equally importantly, the intent of radiotherapy—palliative or curative—is not known and the value of radiotherapy is not clear. Additionally, because pathological sections are not centrally re-confirmed by professional pathologists, CDC is easily misdiagnosed as others, including medullary carcinoma and FH-deficient RCC. These defects are unavoidable. However, this study is of tremendous value to help clinicians comprehend the prognosis of CDCs and make the right treatment strategies for this tumor.

## Conclusion

CDC is an extremely rare renal carcinoma, with an invasive biological behavior. Most patients have a high pathological grade and advanced TNM stage at diagnosis and exhibited poor survival. Larger tumor size, advanced TNM stage, later AJCC stage, and higher pathological grade may indicate an extremely detrimental prognosis. Healthier benefits are more likely to undergo surgery or chemotherapy than more comorbid ones. Certainly, to make systemic therapeutic options for this tumor, long-term large-scale prospective trials are necessary.

## Data Availability Statement

The original contributions presented in the study are included in the article/**Supplementary Material**. Further inquiries can be directed to the corresponding authors.

## Author Contributions

XYQ, JQZ and SGW conceived and designed the study. JZX, and CQL collected and assembled the data. XYQ, MLZ, and SYH, contributed to data processing, interpretation of results, and drafting. XYQ, JNZ, and CQ critically revised the manuscript. All authors read and approved the manuscript.

## Conflict of Interest

The authors declare that the research was conducted in the absence of any commercial or financial relationships that could be construed as a potential conflict of interest.

## Publisher’s Note

All claims expressed in this article are solely those of the authors and do not necessarily represent those of their affiliated organizations, or those of the publisher, the editors and the reviewers. Any product that may be evaluated in this article, or claim that may be made by its manufacturer, is not guaranteed or endorsed by the publisher.

## Abbreviations

CDC, renal collecting duct carcinoma; SEER, Surveillance, Epidemiology, and End Results; OS, overall survival; CSS, cancer-specific survival; DAG, directed acyclic graphs; IQR, interquartile range; HR, hazard ratio; CI, confidence interval; Cox, proportional hazards regression model; TNM, tumor node metastasis; RN, radical nephrectomy; CNx, cytoreductive nephrectomy.
