# Supplementary material for: Impact of Treatment Modalities on Prognosis in Patients With Renal Collecting Duct Carcinoma: A Population-Based Study
Source: Front Oncol. 2022 Apr 22;12:810096. doi: 10.3389/fonc.2022.810096 (PMC9076102; doi:10.3389/fonc.2022.810096)
Supplement: Supplementary file 1 [file DataSheet_1.docx]

Impact of Treatment Modalities on Prognosis in Patients with Renal Collecting Duct Carcinoma: A Population-Based Study

Author name: Xiaoyuan Qian^1^, Jinzhou Xu^1^, Chenqian Liu^1^, Mingliang Zhong^1^, Senyuan Hong^1^, Can Qian^2^, Jianning Zhu^3^, Jiaqiao Zhang^1*†^, Shaogang Wang^1*†^

^1^Author affiliations: Department of Urology, Tongji Hospital, Tongji Medical College, Huazhong University of Science and Technology, Wuhan, China

^2^Author affiliations: Department of Traditional Chinese Medicine and Rheumatology, Southwest Hospital, Army Military Medical University, Chongqing, China

^3^Author affiliations: The Central Hospital of Wuhan, Tongji Medical College, Huazhong University of Science and Technology, Wuhan, Hubei, China

* Correspondence:

Jiaqiao Zhang, E-mail: medzjq@163.com

Shaogang Wang, E-mail: sgwangtjm@163.com

† Jiaqiao Zhang and Shaogang Wang contributed equally to this work.

Address: Department of Urology, Tongji Hospital, 1095 Jiefang Avenue, Wuhan City, Hubei Province, China.

Table

Table S1. Clinicopathologic Characteristics of Renal CDCs

Figure legends

Figure S1. Kaplan-Meier Estimate of Overall Survival (OS) by (A) Tumor size, (B) Pathologic grade, (C) AJCC stage, (D) T stage, (E) N stage, (F) M stage, (G) Surgery, (H) Radiotherapy and (I) Chemotherapy.

Figure S2. Kaplan-Meier Estimate of Cancer-specific survival (CSS) by (A) Tumor size, (B) Pathologic grade, (C) AJCC stage, (D) T stage, (E) N stage, (F) M stage, (G) Surgery, (H) Radiotherapy and (I) Chemotherapy.

Figure S3. Directed Acyclic Graphs (DAG) Showing the Impact of Surgery on Overall Survival (OS) and cancer-specific survival (CSS).

Figure S4. Directed Acyclic Graphs (DAG) Exhibiting the Impact of Chemotherapy on Overall Survival (OS) and cancer-specific survival (CSS).

Figure S5. Directed Acyclic Graphs (DAG) Presenting the Impact of Radiotherapy on Overall Survival (OS) and cancer-specific survival (CSS).

Table S1

| Table S1. Clinicopathologic Characteristics of Renal CDCs | |
| --- | --- |
| Variables | Number (%) |
| Age(years), IQR | 62.0 [52.2, 71.8] |
| Sex |  |
| Female | 71(29.3%) |
| Male | 171(70.7%) |
| Race |  |
| Black | 60(24.8%) |
| White | 166(68.6%) |
| Other | 16(6.6%) |
| Laterality |  |
| Left | 136(56.2%) |
| Right | 106(43.8%) |
| Tumor size(mm), IQR | 62.5 [40.8，85.0] |
| Pathologic grade |  |
| Grade I | 9(3.72%) |
| Grade II | 26(10.7%) |
| Grade III | 92(38.0%) |
| Grade IV | 64(26.4%) |
| Unknown | 51(21.1%) |
| Tumor stage |  |
| I | 57(23.6%) |
| II | 11(4.6%) |
| III | 57(23.6%) |
| IV | 111(45.9%) |
| Unknown | 6(2.3%) |
| T stage |  |
| T1 | 77(31.8%) |
| T2 | 16(6.6%) |
| T3 | 121(50.0%) |
| T4 | 21(8.7%) |
| TX | 7(2.9%) |
| N stage |  |
| N0 | 141(58.3%) |
| N1 | 50(20.7%) |
| N2 | 44(18.2%) |
| NX | 7(2.8%) |
| M stage |  |
| M0 | 152(62.8%) |
| M1 | 86(35.5%) |
| MX | 4(1.7%) |
| Surgery |  |
| Yes | 206(85.1%) |
| None | 36(14.9%) |
| Radiotherapy |  |
| Yes | 25(10.3%) |
| None/Unknown | 217(89.7%) |
| Chemotherapy |  |
| Yes | 64(26.4%) |
| No/Unknown | 178(73.6%) |
|  | |

Figure S1


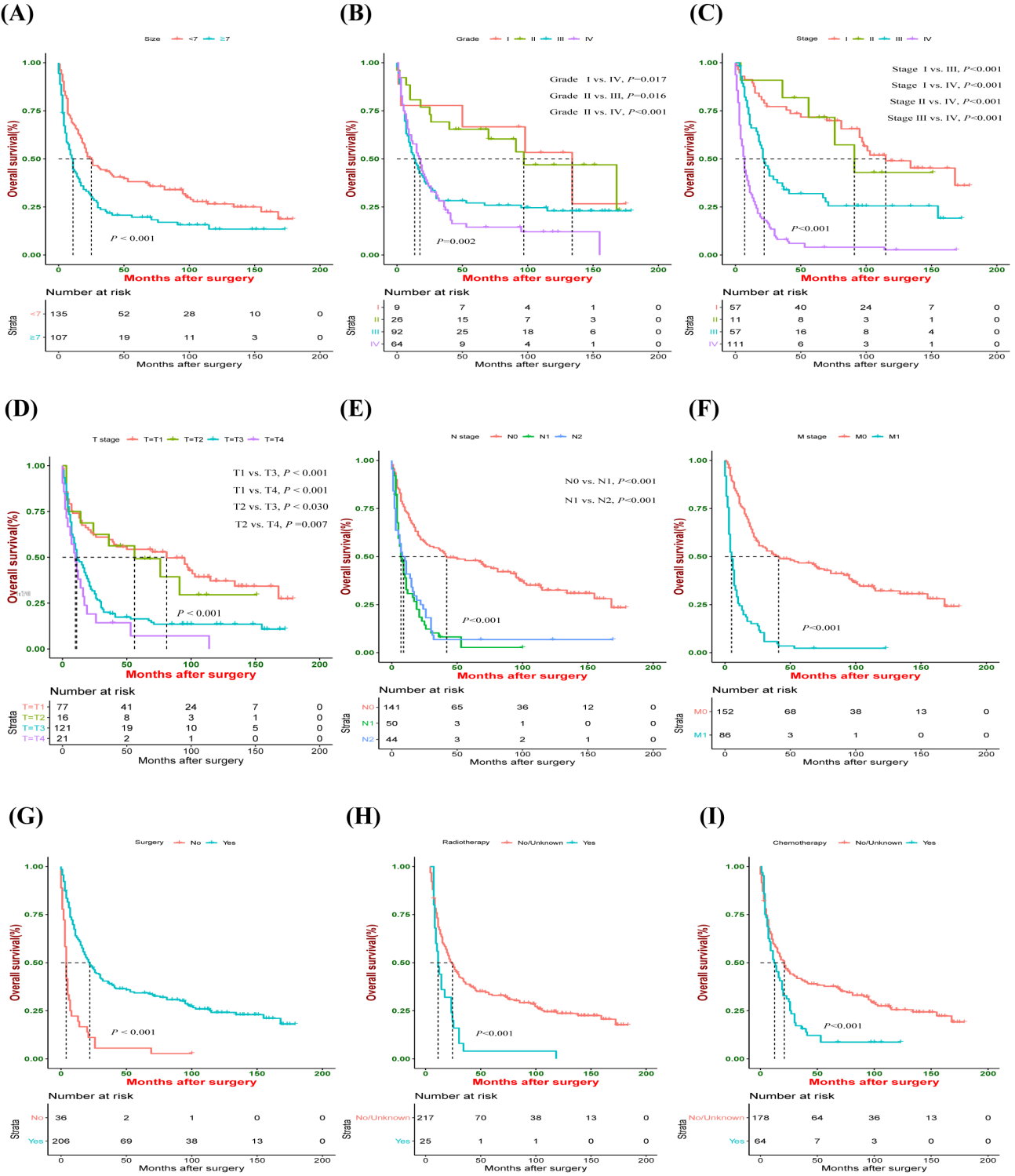


Figure S1. Kaplan-Meier Estimate of Overall Survival (OS) by (A) Tumor size, (B) Pathologic grade, (C) AJCC stage, (D) T stage, (E) N stage, (F) M stage, (G) Surgery, (H) Radiotherapy and (I) Chemotherapy.

Figure S2


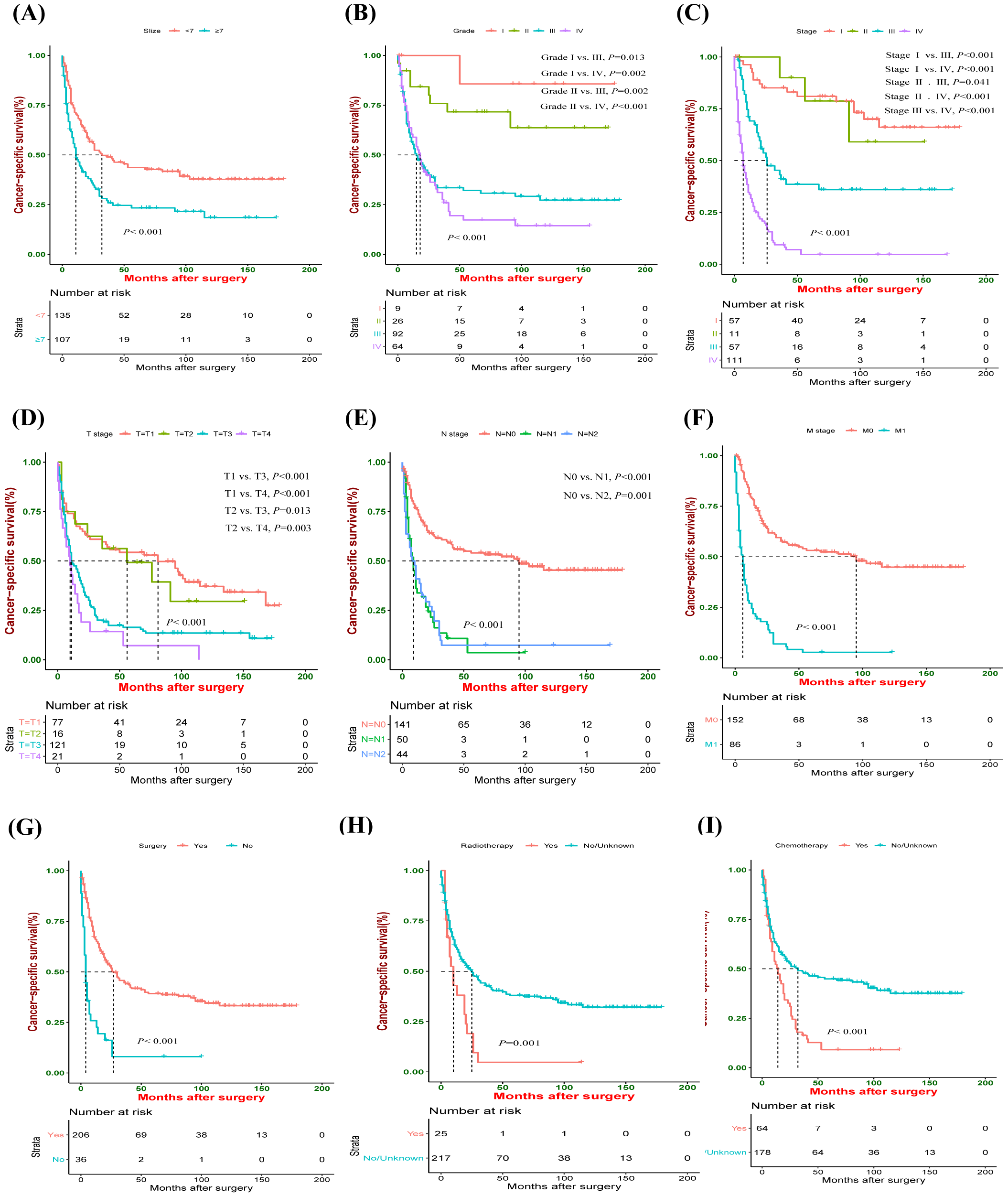


Figure S2. Kaplan-Meier Estimate of Cancer-specific survival (CSS) by (A) Tumor size, (B) Pathologic grade, (C) AJCC stage, (D) T stage, (E) N stage, (F) M stage, (G) Surgery, (H) Radiotherapy and (I) Chemotherapy.

Figure S3


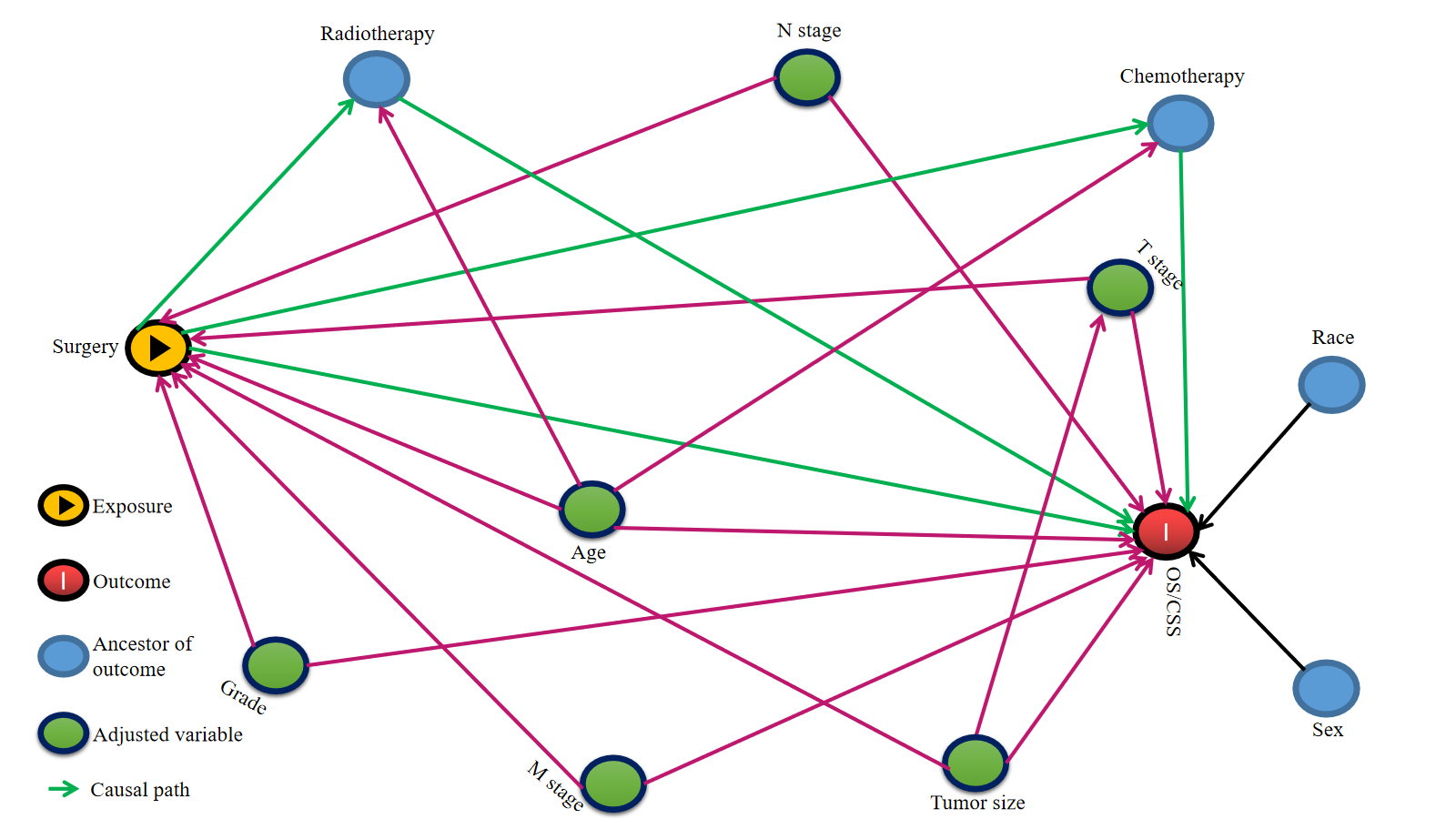


Figure S3. Directed Acyclic Graphs (DAG) Showing the Impact of Surgery on Overall Survival (OS) and cancer-specific survival (CSS).

Figure S4


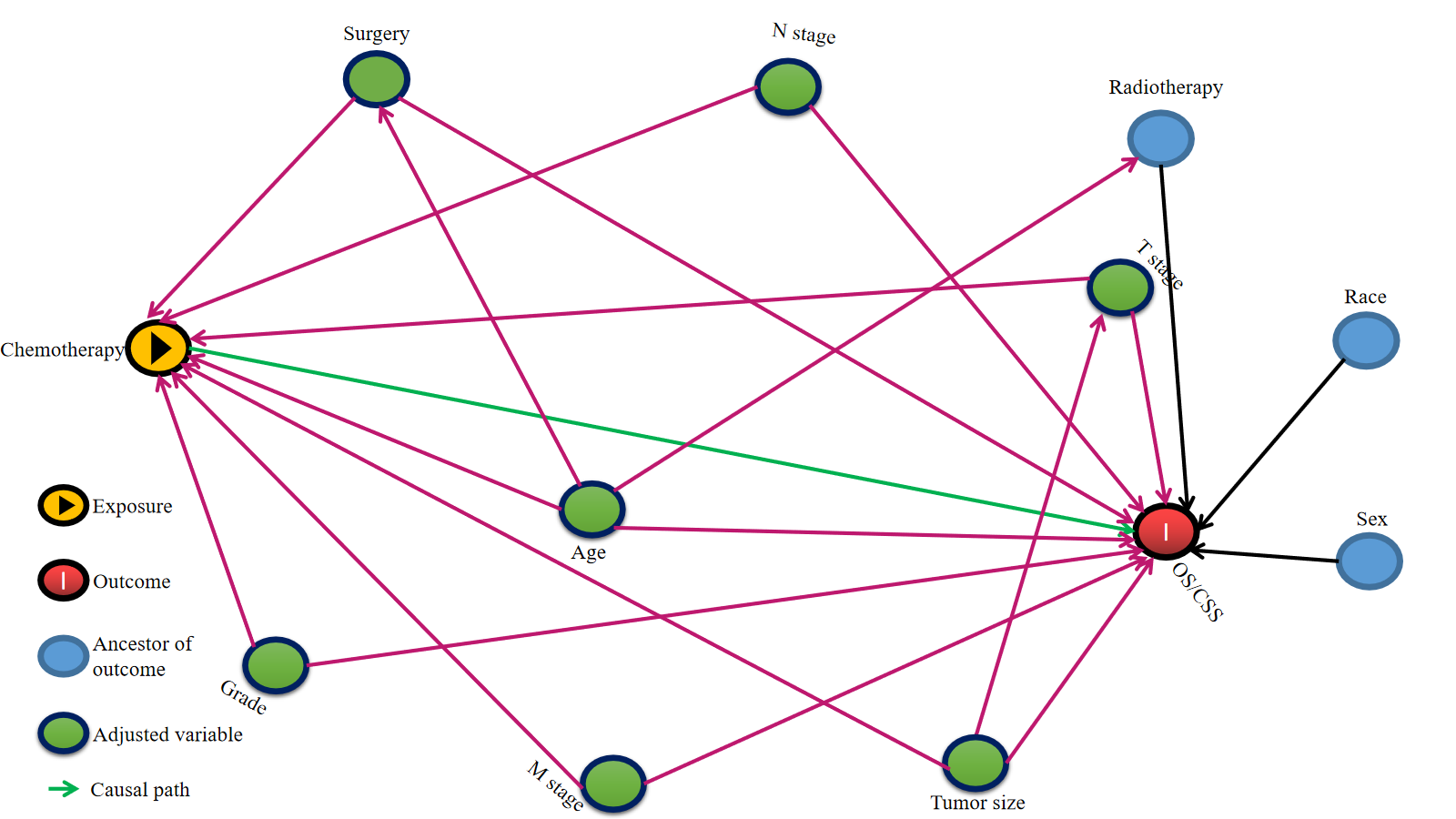


Figure S4. Directed Acyclic Graphs (DAG) Exhibiting the Impact of Chemotherapy on Overall Survival (OS) and cancer-specific survival (CSS).

Figure S5


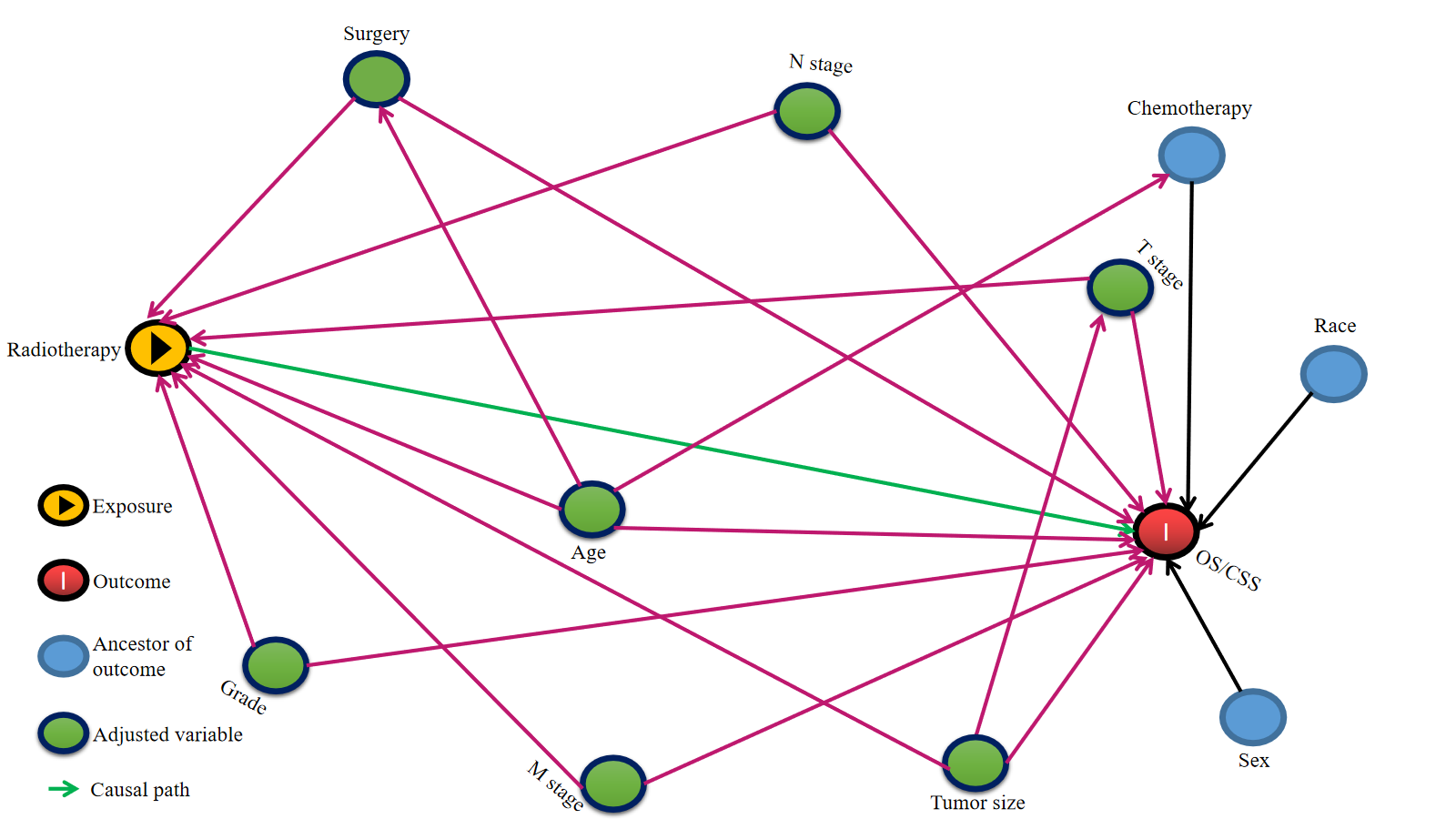


Figure S5. Directed Acyclic Graphs (DAG) Presenting the Impact of Radiotherapy on Overall Survival (OS) and cancer-specific survival (CSS).
